# A Phthalimide-Functionalized Heptamethine Cyanine Dye for Tumor-Targeted Photothermal Therapy

**DOI:** 10.3390/cancers16244155

**Published:** 2024-12-13

**Authors:** Yoonbin Park, Juhui Yang, Hoon Hyun

**Affiliations:** 1 Department of Biomedical Sciences, Chonnam National University Medical School, Hwasun 58128, Republic of Korea; unb1n0213@naver.com (Y.P.); joooh1008@gmail.com (J.Y.); 2 BioMedical Sciences Graduate Program (BMSGP), Chonnam National University, Hwasun 58128, Republic of Korea

**Keywords:** heptamethine cyanine dyes, near-infrared fluorescence imaging, photothermal therapy, tumor-targeted imaging, structure-inherent targeting

## Abstract

Background: A phthalimide-functionalized heptamethine cyanine dye, named Ph790H, is used for targeted photothermal cancer therapy in vivo. We highlight that the chemical structure of Ph790H is newly designed and synthesized for the first time in this study. Objectives: By possessing a rigid chloro-cyclohexenyl ring in the heptamethine cyanine backbone, the bifunctional near-infrared (NIR) fluorescent dye Ph790H can be preferentially accumulated in tumor without the need for additional targeting ligands, which is defined as the “structure-inherent tumor targeting” concept. Methods: The phototherapeutic effect of Ph790H is evaluated in HT-29 human colorectal cancer xenografts to be used as a cancer-targeting photothermal agent. Results: The results reveal that the Ph790H shows enhanced tumor accumulation in HT-29 xenografts 48 h post-injection with a high tumor-to-background ratio. After determination of the optimal timing for photothermal therapy (PTT), the HT-29 tumor-possessing nude mice pretreated with Ph790H are subsequently irradiated with an 808 nm NIR laser for 5 min. The tumor-targeted PTT treatment can efficiently inhibit the tumor development compared with that of control groups. Moreover, no tumor regrowth or Ph790H-induced mortality occurs after the treatment of Ph790H and laser irradiation during a period of monitoring. Conclusions: Therefore, this work demonstrates that the bifunctional phototheranostic agent Ph790H can be utilized for targeted cancer imaging and fluorescence-guided phototherapy simultaneously.

## Simple Summary

Most commercial heptamethine cyanine dyes have no tumor targetability owing to their limited intrinsic selectivity. The development of multifunctional heptamethine cyanine dyes that can enable both tumor-specific imaging and therapy is highly desirable for simple and effective cancer treatments. For this reason, we newly synthesized a tumor-targetable heptamethine cyanine dye which not only showed enhanced accumulation in the tumor site, but was also highly effective as an in vivo photothermal cancer therapy in a HT-29 human colon cancer xenograft mouse model. On the principle of the “structure-inherent targeting” approach, a single-molecule NIR dye could achieve tumor targeting, imaging, and therapy simultaneously. Hence, the present study suggests a simple but efficient strategy to design and synthesize the multifunctional small-molecule theranostic agents for tumor-targeted imaging and phototherapy.

## 1. Introduction

In recent years, photothermal therapy (PTT) combined with tumor-targeted near-infrared (NIR) fluorescent dyes has attracted much attention because it allows for fluorescence-guided real-time and in situ therapy [1,2,3]. PTT is a promising modality for effective cancer therapy owing to its intrinsic advantages in terms of cost, convenience, and safety with high tumor ablation efficiency [4,5,6]. Upon NIR light irradiation, the NIR fluorescent dyes, which are one type of a diverse range of PTT agents, convert the incident light into heat energy to physically damage the tumor cells. Most of the cyanine-based PTT agents are widely used in the NIR window (700–900 nm) because the multifunctional polymethine cyanine dyes have been continually developed for targeted cancer imaging and PTT/photodynamic therapy (PDT) by mostly using 671 and 808 nm laser sources [7,8,9]. The two most common laser-based phototherapies utilize light and photo-absorbing agents to produce cytotoxic reactive oxygen species or a local temperature increase, respectively. Since PDT application in cancer treatment has several limitations, mainly due to tumor hypoxia, PTT has attracted more attention owing to oxygen independence.

Importantly, among the various polymethine cyanine dyes developed so far, which mainly consist of heptamethine or pentamethine cyanine skeletons, the heptamethine cyanine structure having a chloro-cyclohexenyl ring is much more favorable than other types of cyanine dyes in terms of in vivo tumor targetability enhanced by the formation of albumin adducts [10,11,12]. On the other hand, the electron-withdrawing chloro-cyclohexenyl ring pulls electrons from the heptamethine chain to readily undergo nucleophilic substitution through a S_RN_1 mechanism to replace the central chlorine atom with diverse functional groups, including alkyl-ether, aryl-ether, and aryl-amine linkages, for the conjugation of various biomolecules [8]. Additionally, it was previously reported that the commercial heptamethine cyanine dyes, including IR-780, IR-783, IR-786, and IR-820, possessing a rigid heptamethine backbone with the chloro-cyclohexenyl ring exhibited not only enhanced photostability and quantum yield, but also reduced photobleaching and aggregation behavior compared with that of indocyanine green (ICG) consisting of a heptamethine chain without the chloro-cyclohexenyl ring, which is the FDA-approved NIR fluorescent dye used for clinical diagnostic applications [8]. Thus, the heptamethine cyanine dyes containing the chloro-cyclohexenyl bridge are highly suitable for NIR fluorescence imaging and phototherapy applications.

Most heptamethine cyanine dyes available commercially have no tumor targetability. The development of multifunctional heptamethine cyanine dyes that can enable both tumor-specific imaging and therapy is highly desirable for simple and effective cancer treatments. Since the ICG has no chemical groups used for conjugation to other molecules, the various types of conjugatable heptamethine cyanine dyes, including Cy7, IRDye800CW, IR-808, and ZW800-1, have been commercially available for multipurpose NIR fluorescence imaging and other applications [13,14,15]. However, Choi et al. suggested that the targeting efficiency and therapeutic efficacy of the conjugates may be altered or reduced by the physicochemical properties (e.g., hydrophobicity, polarity, molecular conformation, and net surface charge) of conjugated NIR dyes [16].

An advanced strategy to overcome this limitation in target-specific NIR fluorescence imaging has been suggested to integrate the three functions (i.e., target specificity, fluorescence emission, and/or therapeutic potential) into a single-molecule NIR dye through the concept of “structure-inherent targeting” [17,18,19]. So far, various types of multifunctional heptamethine cyanine dyes, which showed tumor uptake natively without the conjugation to tumor-targeting ligands, have been designed and synthesized to be used for tumor-targeted fluorescence imaging [20,21,22]. Further, the tumor-targeted heptamethine cyanine dyes occasionally revealed potent therapeutic efficacy in various types of cancer. The key consideration for the design of multifunctional heptamethine cyanine dyes using this structure-inherent targeting strategy is the modulation of chemical structures (i.e., the heptamethine chain for PTT, indole side chains for the tumor-targeting domain, and *N*-alkyl side chains for chemotherapy).

In this study, we demonstrated the photothermal efficacy of the newly designed heptamethine cyanine dye, named Ph790H, combined with NIR laser irradiation in in vitro and in vivo studies using HT-29 cancer cells. The in vivo results revealed that Ph790H not only displayed enhanced and prolonged tumor accumulation in HT-29 xenografts but also exhibited superior photothermal conversion efficiency after laser irradiation for complete tumor ablation. Therefore, this work demonstrates that the bifunctional phototheranostic agent Ph790H can be utilized for targeted cancer imaging and fluorescence-guided phototherapy simultaneously.

## 2. Materials and Methods

### 2.1. Synthesis of the Heptamethine Cyanine Dye Ph790H

All chemicals and solvents were purchased from Sigma-Aldrich (St. Louis, MO, USA). 2,3,3-trimethylindolenine **3** (0.7 g, 4.3 mmol) and N-(3-bromopropyl)phthalimide **4** (1.34 g, 5 mmol) were mixed in toluene (50 mL) and heated at 100 °C for 72 h under a nitrogen atmosphere. After reaction, the mixture was washed with toluene several times to remove unreacted compounds. The solid was collected and used directly in the next step (0.76 g, 51.2%). Subsequently, compound **5** (0.76 g, 2.2 mmol), Vilsmeier-Haack reagent **6** (0.32 g, 1 mmol), and anhydrous sodium acetate (0.25 g, 3 mmol) were mixed in absolute ethanol (20 mL) and heated under reflux for 6 h under a nitrogen atmosphere. After the reaction, the residue was dissolved in dimethyl sulfoxide (DMSO) and purified by a preparative high-performance liquid chromatography (HPLC) system (Waters, Milford, MA, USA). The final compound Ph790H was collected as a dark green solid (0.68 g, 82% yield with 97% purity). The obtained Ph790H was confirmed with the Dionex UltiMate^TM^ 3000 mass spectrometry system (Thermo Scientific, Waltham, MA, USA). The accurate mass HRMS (ESI) *m*/*z* [M]^+^ was calculated for [C_52_H_50_ClN_4_O_4_]^+^ 829.3515, and found [M]^+^ 829.3500.

### 2.2. Optical and Physicochemical Property Analyses

Fetal bovine serum (FBS, pH 7.4) (Welgene, Gyeongsan, Republic of Korea) was used to dissolve Ph790H. A fiber optic FLAME spectrometer (Ocean Optics, Dunedin, FL, USA) was used to measure the absorbance of Ph790H. The Beer–Lambert equation was used to determine the molar extinction coefficient (*ε*) of Ph790H. A SPARK^®^ 10M microplate reader (Tecan, Männedorf, Switzerland) was used to analyze the fluorescence emission spectrum excited at 740 nm. Indocyanine green (ICG) dissolved in DMSO (*Φ* = 13% as a calibration standard) was used to determine the fluorescence quantum yield (*Φ*) of Ph790H by matching the absorbance at 770 nm [16]. Marvin and JChem calculator plugins (under version 14.12.15.0, ChemAxon, Budapest, Hungary) were used to analyze in silico predictions of the distribution coefficient (log*D* at pH 7.4) and topological polar surface area (TPSA).

### 2.3. In Vitro Cytotoxicity Assay

The human large-cell lung carcinoma cell line (NCI-H460), breast adenocarcinoma cell lines (MDA-MB-231 and MCF-7), colorectal adenocarcinoma cell line (HT-29), and mouse embryonic fibroblast cell line (NIH/3T3) were purchased from the American Type Culture Collection (ATCC; Manassas, VA, USA). Dulbecco’s Modified Eagle’s Medium (DMEM) or Roswell Park Memorial Institute (RPMI) 1640 media containing FBS, penicillin, streptomycin, and amphotericin B (Welgene) were used to maintain cultured cells. The cytotoxicity of Ph790H was examined using the 3-(4,5-dimethylthiazol-2-yl)-2,5-diphenyltetrazolium bromide (MTT) assay. Cells were seeded onto 96-well plates (1 × 10^4^ cells per well). To evaluate the cytotoxicity depending on the concentrations of each sample, the cells were treated with Ph790H (2, 10, 20, and 50 μM) for 4 h and cultured for 24 h after treatment. At each time point, the incubation cell medium was replaced with 100 μL of fresh medium, and 10 μL of the MTT solution was directly added to each 100 μL well. Subsequently, the plates were then incubated for 4 h at 37 °C in a humidified 5% CO_2_ incubator. Finally, the plates were placed in a microplate reader (SPARK^®^ 10M, Tecan) to measure the absorption intensity at 570 nm. Cell viability was calculated using the following formula: cell viability (%) = (*A*_sample_ − *A*_blank_)/(*A*_control_ − *A*_blank_) × 100, where *A* is the average absorbance.

### 2.4. In Vitro Live-Cell Imaging

In total, 2 μM of Ph790H was added to HT-29 cells and stored at 37 °C in a humidified 5% CO_2_ incubator for 1 h. After washing with phosphate-buffered saline (PBS, pH 7.4), the cells were observed by a 4-filter set of the Nikon Eclipse Ti-U inverted microscope system (Nikon, Seoul, Republic of Korea).

### 2.5. In Vitro Photothermal Conversion Efficiency

An NIR laser diode system (NaKu Technology Co., Ltd., Hangzhou, China) was used for irradiation of model substrates and mice tumors. The emission wavelength as provided by the supplier was 808 ± 5 nm. The emitted power and beam diameter were ~1000 mW and ~5 mm, respectively. In all cases, the distance between the laser output and the different irradiated targets was kept constant at 10 cm. Ph790H (50 μM) dissolved in FBS (100 μL, pH 7.4) was irradiated with an 808 nm laser (1.0 W/cm^2^). A thermal imager (FLIR Systems, Wilsonville, OR, USA) was used to monitor temperature change in real time. The photothermal stability of Ph790H was tested by repeating the heating and cooling cycle three times. The photothermal conversion efficiency (*η*) of Ph790H was calculated based on the equations reported previously [23].

### 2.6. In Vitro Photothermal Cytotoxicity

Ph790H (10 µM) was added to HT-29 cancer cells and incubated for 4 h. After washing with PBS, the cells were irradiated with the 808 nm laser (1.0 W/cm^2^) for 1 min. Subsequently, Calcein-AM and propidium iodide (PI; Thermo Fisher Scientific) were used to co-stain the cells for 30 min. After washing with PBS, the fluorescent microscope (Nikon) was used to confirm the stained cells.

### 2.7. HT-29 Xenograft Mouse Model

The animal experiment was approved by the Chonnam National University Animal Research Committee (CNU IACUC-H-2023-57). Male nude mice (6 weeks old, ≈25 g, 3 mice in a Ph790H-injected group used for time-dependent tumor imaging, and a total of 9 mice in three different treatment groups used for PTT treatment) were purchased from OrientBio (Gwangju, Republic of Korea). An HT-29 xenograft was prepared by the inoculation of HT-29 cancer cells (1 × 10^6^ cells per mouse) subcutaneously into the right flank of each mouse. At 10 days post-inoculation, Ph790H was dissolved in a saline solution containing 5% wt./v bovine serum albumin (BSA) and injected intravenously.

### 2.8. In Vivo Time-Dependent Tumor Imaging

The FOBI fluorescence imaging system (NeoScience, Deajeon, Republic of Korea) was used to monitor the tumor-bearing mice in real time. The mice (3 mice in the PBS or Ph790H treatment group) were monitored for 72 h after intravenous injection of Ph790H. The tumor fluorescence intensity was measured using ImageJ software (National Institutes of Health, Bethesda, MD, USA, https://imagej.net/ij/, accessed on 1 October 2024). A thermal imager (FLIR Systems) was also used to monitor the tumor temperature for 5 min.

### 2.9. In Vivo Photothermal Therapeutic Efficacy

The HT-29 xenograft mouse model was used to inject the Ph790H or PBS (3 mice in the PBS with laser, Ph790H alone, or Ph790H with laser treatment group). At 48 h post-injection, the tumor area of each mouse was subjected to irradiate the 808 nm laser (1.0 W/cm^2^) for 5 min. The temperature changes at the tumor site were observed using the thermal imager (FLIR Systems). Subsequently, the mice in each group were sacrificed to harvest the tumors for the histological confirmation using the hematoxylin and eosin (H&E) staining. In addition, mice were monitored to observe tumor development and body weight for 11 days. The calculation formula V = 0.5 × longest diameter × (shortest diameter)^2^ was used to measure the tumor volume (V).

### 2.10. Histological Analysis

Tumor samples (*n* = 3) were collected from each mouse group and stored for H&E staining. The tumors were fixed in 4% paraformaldehyde and flash frozen in an optimal cutting temperature (OCT) compound using liquid nitrogen. Frozen samples were cryosectioned (10 µM thick), stained with H&E, and observed using a microscope. Histological confirmation of each tumor section was carried out using the Nikon Eclipse Ti-U inverted microscope system.

### 2.11. Statistical Analysis

Statistical analysis was determined by a one-way analysis of variance followed by Tukey’s multiple comparison test using the Prism software version 5.01 (GraphPad, San Diego, CA, USA). The results were represented as mean ± standard deviation (S.D.), and a *p*-value less than 0.05 was statistically significant.

## 3. Results

### 3.1. Synthesis and Characterization of Ph790H

**Figure 1:**
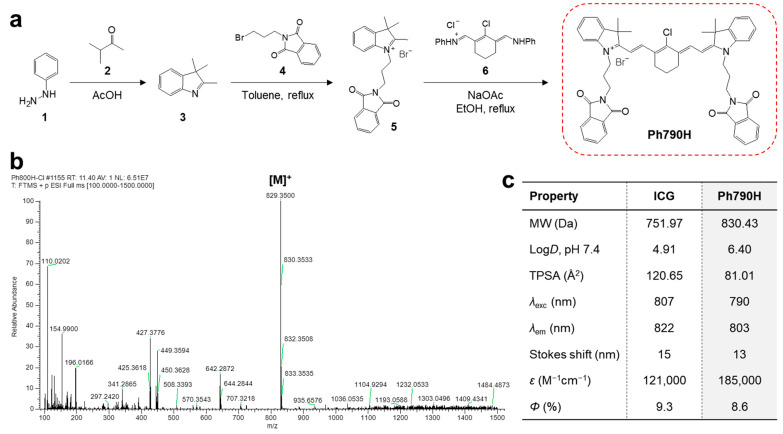
(**a**) Synthetic process and (**b**) mass data of Ph790H. (**c**) Physicochemical and optical properties of ICG [24] and Ph790H.

A phthalimide-functionalized heptamethine cyanine dye Ph790H was prepared according to the synthesis and purification process reported previously [24,25]. The heptamethine cyanine backbone was synthesized through the condensation reaction between phthalimide-functionalized heterocyclic salts **5** and the Vilsmeier–Haack reagent **6** (Figure 1a). After purification, the molecular weight of Ph790H was analyzed using mass spectrometry to confirm the successful synthesis of Ph790H (Figure 1b). As summarized in Figure 1c, the peak absorption at 790 nm and maximum fluorescence emission at 803 nm for the Ph790H dye dissolved in serum were detected with a typical Stokes shift of 13 nm. Ph790H exhibited a moderate molar extinction coefficient (*ε* = 185,000 M^−1^cm^−1^) and quantum yield (*Φ* = 8.6%) measured in serum, compared with that of ICG available clinically. Ph790H displayed a relatively higher hydrophobicity (log*D* = 6.40) and lower polarity (TPSA = 81.01 Å^2^), as compared with that of ICG.

### 3.2. In Vitro Cytotoxicity, Cell Binding, and Photothermal Effect

**Figure 2:**
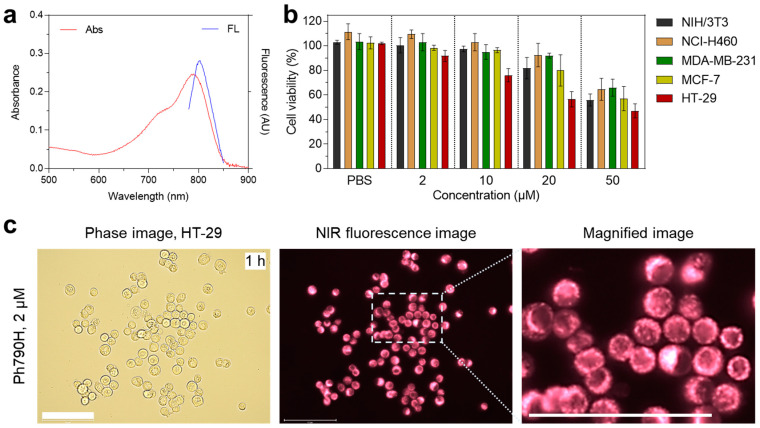
(**a**) Absorbance and fluorescence of Ph790H measured in serum. (**b**) Cytotoxicity analysis of Ph790H using NIH/3T3, NCI-H460, MDA-MB-231, MCF-7, and HT-29 cells. (**c**) Cell binding of Ph790H in HT-29 cells. Scale bars = 100 μM.

The absorption and fluorescence emission spectra of Ph790H were measured in serum due to its high hydrophobicity (Figure 2a). The cytotoxicity of Ph790H was found using the MTT assay in NIH/3T3, NCI-H460, MDA-MB-231, MCF-7, and HT-29 cells after the treatment of Ph790H (2–50 μM) (Figure 2b). To check the cytotoxic effect on normal cells, NIH/3T3 fibroblasts were treated with Ph790H under the same conditions. The NIH/3T3 cells showed no obvious cytotoxicity between 0 and 20 μM concentrations of Ph790H. Also, the NCI-H460, MDA-MB-231, and MCF-7 cancer cells showed no significant cytotoxicity in the concentration range of 0–20 μM of Ph790H, which is similar to that of NIH/3T3 cells. Interestingly, the cytotoxicity of HT-29 cancer cells treated with Ph790H increased depending on the concentrations. This result indicates that Ph790H has cell type-specific toxicity and can be used for the treatment of colon cancer. Moreover, we observed the cell binding of Ph790H after incubation in HT-29 cancer cells for 1 h (Figure 2c). As expected, Ph790H exhibited significant intracellular localization in the cytoplasm with high fluorescence intensity. This result indicates that Ph790H can deliver the photothermal energy into the HT-29 cancer cells during NIR laser irradiation for inducing cancer cell death.

**Figure 3:**
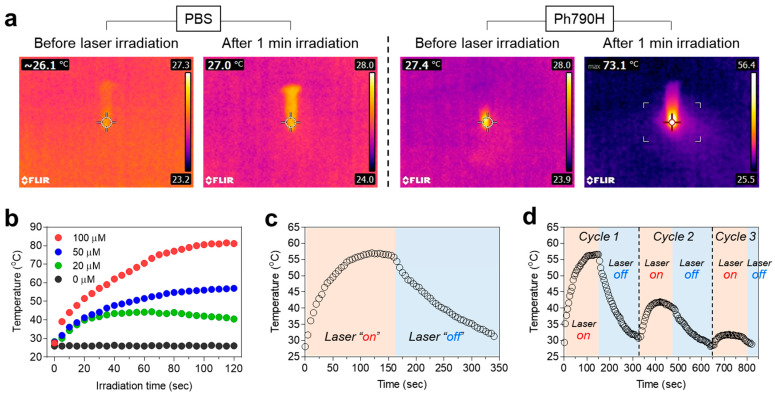
(**a**) Thermal images of PBS and Ph790H (100 μM) solutions before and after laser irradiation for 1 min. (**b**) Temperature changes in PBS and Ph790H (20, 50, and 100 μM) solutions were monitored for 120 s under laser irradiation. (**c**) Heating and cooling curve of Ph790H (50 μM) under laser irradiation. (**d**) Three on/off cycles of the Ph790H (50 μM) solution.

After confirming cytotoxicity and intracellular localization of Ph790H, the photothermal conversion efficiency of Ph790H was examined by analyzing the temperature changes in Ph790H (100 μM in serum) under 808 nm laser irradiation (1.0 W/cm^2^) for 1 min. The thermal imager was used in real time to observe the temperature variation. The temperature of the Ph790H solution become elevated immediately, reaching up to 73.1 °C, after 1 min of irradiation, whereas the PBS solution exhibited little change in temperature after 1 min of irradiation (Figure 3a). Also, we examined the temperature changes in different concentrations of Ph790H upon 808 nm laser irradiation for 2 min. As expected, the Ph790H solutions showed a concentration-dependent increase in temperature after 2 min of irradiation (Figure 3b). Based on the temperature variation in the Ph790H solution displayed in Figure 3c, the photothermal conversion efficiency (*η*) of Ph790H was determined to be 31.1%, which is similar to that of the cyanine dyes reported previously [13,26,27]. This experiment demonstrates that Ph790H is suitable for photothermal tumor therapy in vivo. Additionally, we tested the photothermal stability of Ph790H by using the heating and cooling cycle (150 sec heating followed by 200 s cooling for each cycle). As shown in Figure 3d, the photostability of Ph790H in serum obviously declined during the second cycle. Moreover, the solution temperature of Ph790H was largely diminished during the third cycle of laser irradiation. This experiment result indicates that the heptamethine cyanine skeleton can irreversibly be damaged by irradiating the concentrated NIR light.

**Figure 4:**
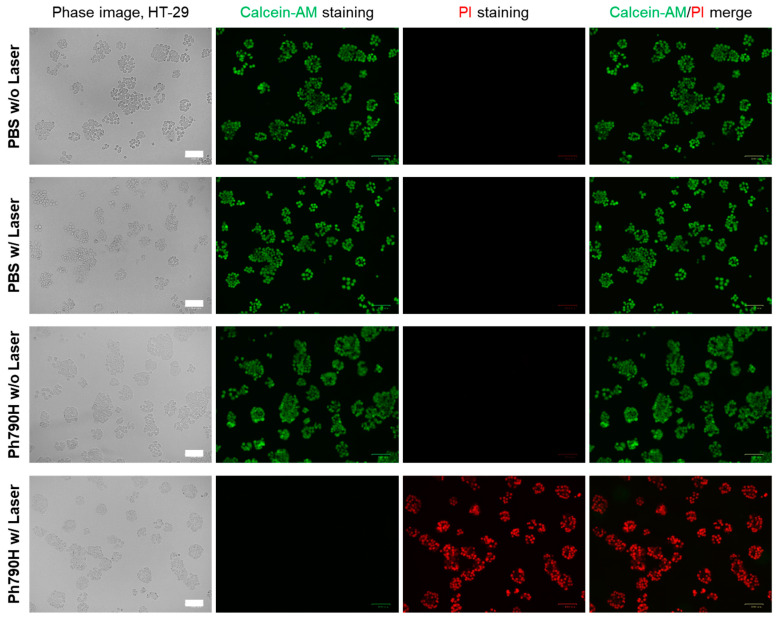
Fluorescence images of HT-29 cells treated with or without PTT treatment. Calcein-AM (green color) and propidium iodide (red color) were used to stain the HT-29 cells after each treatment. Scale bars = 100 μm.

To observe the phototoxicity of HT-29 cells under laser irradiation, Calcein-AM (green for live cells) and propidium iodide (PI, red for dead cells) were used to stain HT-29 cancer cells before and after laser irradiation, respectively. The cancer cells were pretreated with Ph790H for 4 h and then exposed to 808 nm laser irradiation (1.0 W/cm^2^) for 1 min. As shown in Figure 4, the HT-29 cancer cells treated with the combination of Ph790H and laser irradiation showed extensive cell death compared to other treatment groups (i.e., PBS without laser, PBS with laser, and Ph790H without laser irradiation), which are clearly confirmed by the PI and Calcein-AM staining. Thus, this result demonstrates that Ph790H (10 μM) alone or laser alone displayed no significant effect on cancer cell death. In this respect, high tumor accumulation of Ph790H is the most important requirement for maximizing the PTT effect in vivo.

### 3.3. Time-Dependent In Vivo Tumor Retention and Photothermal Effect

**Figure 5:**
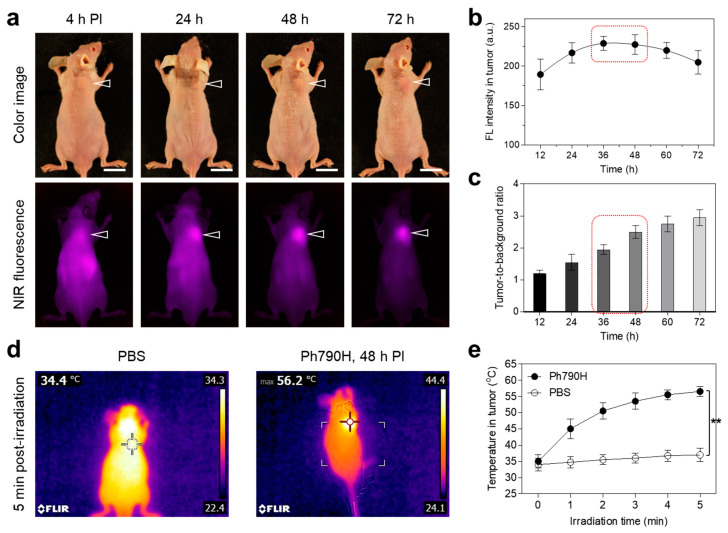
(**a**) In vivo NIR fluorescence imaging of HT-29 xenografts after injection of Ph790H. Arrowheads indicate the tumor area. Scale bars = 1 cm. (**b**) Fluorescence intensity and (**c**) tumor-to-background ratio at the tumor area for 72 h. (**d**) Thermal images and (**e**) temperature changes in each mouse group at the tumor area. Data are expressed as mean ± S.D. (*n* = 3). ** *p* < 0.01.

After confirming the effective cell phototoxicity of the combination of Ph790H and laser irradiation, we investigated the tumor-targeting efficiency of Ph790H using a HT-29 xenograft mouse model. The HT-29 xenografts were treated with Ph790H (0.6 mg/kg) and dissolved in a BSA solution through an intravenous administration. Remarkably, Ph790H showed high tumor accumulation over 72 h post-injection (Figure 5a). The tumor fluorescence intensity was continually elevated up to 48 h post-injection and gradually decreased after 72 h post-injection (Figure 5b). To find the optimal timing of laser irradiation, we determined the best time with high signals in the tumor but low signals in the surrounding tissues based on the tumor-to-background ratio over time (Figure 5c). The values of the tumor-to-background ratio were continuously elevated up to 72 h post-injection, owing to the maintained fluorescence signals in the tumor and decreased fluorescence signals in the surrounding tissues. Considering correlation between the tumor site and neighboring tissues, we carried out the laser irradiation on the tumor area at 48 h post-injection to avoid unnecessary damage to neighboring normal tissues.

Subsequently, we performed the 808 nm laser irradiation (1.0 W/cm^2^) for 5 min using the HT-29 xenografts at 48 h post-injection of Ph790H. The power density of the NIR laser was optimized previously to prevent the normal tissue damage affected by the laser irradiation alone [28]. Interestingly, the temperature in the tumors treated with Ph790H and laser irradiation increased up to 56.2 °C during 5 min of laser irradiation, while the tumors injected with PBS exhibited no significant change in the temperature under the laser irradiation condition (Figure 5d). The temperature in tumors treated with Ph790H was gradually elevated by approximately 50 °C after 2 min of laser irradiation, and then the tumor temperature was maintained up to 55 °C for the next 3 min of laser irradiation (Figure 5e). These results demonstrate that the temperature (~55 °C) in tumors treated with the combination of Ph790H and laser irradiation is high enough to induce tumor necrosis for complete tumor ablation.

### 3.4. In Vivo Photothermal Therapeutic Efficacy

**Figure 6:**
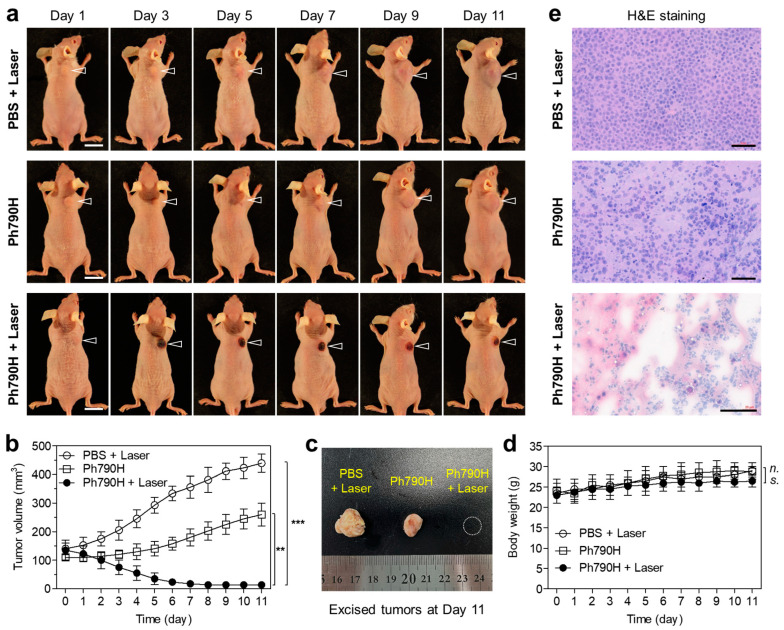
(**a**) Photographs of changes in tumor volume in HT-29 xenografts for 11 days. Arrowheads indicate the tumor area. Scale bars = 1 cm. (**b**) Tumor volumes, (**c**) a photograph of tumors collected from each mouse group at day 11, and (**d**) body weights were measured for 11 days. Data are expressed as mean ± S.D. (*n* = 3). ** *p* < 0.01; *** *p* < 0.001. (**e**) H&E staining of tumor sections collected from each mouse group (*n* = 3). Scale bars = 50 μm.

To evaluate the phototherapeutic effect of Ph790H in vivo, HT-29 xenografts were continuously observed for 11 days after treatment with Ph790H and laser irradiation (Figure 6a). As expected, the tumors treated with PBS and laser irradiation showed no therapeutic effect with a typical growth rate at the tumor site. Interestingly, the tumors treated with Ph790H alone exhibited slow development for the first 5 days after treatment, but the tumors began to grow over the next few days. This result suggests that the phthalimide moiety of Ph790H could inhibit the tumor growth for the early stage of treatment owing to its pharmacophore. More importantly, the tumors treated with the combination of Ph790H and laser irradiation showed a complete tumor ablation without tumor regrowth for 11 days (Figure 6b). Thus, these results demonstrate that Ph790H combined with laser irradiation can effectively ablate tumors based on the photothermal cell death. At day 11, a photograph of tumors collected from each mouse group displayed the obvious differences in tumor size (Figure 6c). Moreover, mice in each group showed no significant loss of body weight for 11 days, which indicates little systemic Ph790H toxicity (Figure 6d). As displayed in Figure 6e, the tumor section treated with PBS with laser irradiation showed normal shapes of cell proliferation. Although the tumor section treated with Ph790H alone exhibited little signs of apoptosis, the tumor section treated with Ph790H and laser irradiation showed apparent morphologic changes. This result suggests that the tumor-targetable Ph790H could be a safe and effective PTT agent for cancer phototherapy.

## 4. Discussion

Most studies have been focused mostly on nanomaterial-based systems for “all-in-one” theranostics by using relatively complicated methods. Although many different types of nanocarriers, including noble metal-, carbon-, protein-, and synthetic polymer-based nanomaterials, have been extensively developed for cancer-targeted chemo- and/or phototherapy, this approach is highly dependent on the tumor targetability of nanocarriers. That is, the size control and surface modification of nanomaterials are essentially needed to improve tumor accumulation and retention as well as clearance and excretion from the body for preventing systemic toxicity. However, there are still limitations to using the nanoparticles, such as complex synthetic processes for obtaining homogeneous products and unsolved biological safety [5]. In this regard, the small-molecular cyanine dyes have recently attracted attention because of their excellent optical and physicochemical properties including noninvasive deep tissue imaging, tumor-specific uptake, and photothermal/photodynamic effect that can be used for simultaneous cancer-targeted imaging and therapy.

There are two major theories reported previously to explain how the heptamethine cyanine dyes can be accumulated in the tumor via the concept of “structure-inherent tumor targeting”. Typically, a well-known mechanism is that the heptamethine cyanine dyes can be taken up by the organic anion transporting polypeptide (OATP) transporters into cancer cells [19]. On the other hand, Usama et al. recently suggested that the formation of covalent or noncovalent albumin adducts between them is a key trapping mechanism in tumors through albumin-mediated endocytosis [10,11,12]. Additionally, the albumin-mediated uptake in tumors is involved with the albumin binding proteins, including secreted protein acidic and rich in cysteine (SPARC) and membrane-associated glycoprotein, as well as the enhanced permeation and retention (EPR) effect [29]. Based on this theory, we understand that the rigid chloro-cyclohexenyl ring on the heptamethine backbone that forms the covalent albumin adducts plays an important role in tumor preferential accumulation of Ph790H.

The phthalimide group is known as a pharmacophore in medicinal chemistry owing to its antimicrobial, antioxidant, anticancer, and anti-inflammatory activities. The phthalimide group consisting of an isoindoline-1,3-dione core with an imide ring enables its movement across biological membranes in vivo. To date, many phthalimide derivatives have been developed and extensively investigated to confirm their therapeutic efficacy [30]. Interestingly, the phthalimide-functionalized heptamethine cyanine dye Ph790H showed delayed tumor development in the early stage of treatment for 5 days after a single-dose injection (0.6 mg/kg), compared to the control group. This suggests that the phthalimide moiety of Ph790H could contribute to its tumor suppressive activity in the early period. Based on this finding, the optimal dose of Ph790H could be made more effective in cancer chemotherapy. This can be further investigated in the next study, because PTT alone without a chemotherapeutic effect is still a limitation to carefully monitor and accurately treat primary tumor development and metastatic activity in orthotopic tumor models. Most importantly, the chemical structure of Ph790H may be a clue to provide a feasible approach for the development of multifunctional polymethine cyanine dyes enabling synergistic chemodynamic, photothermal, and photodynamic therapy.

## 5. Conclusions

In conclusion, we designed and synthesized a tumor-targetable heptamethine cyanine dye Ph790H which not only showed enhanced tumor accumulation 48 h post-injection but also excellent PTT efficacy in a HT-29 xenograft mouse model. On the basis of the “structure-inherent tumor targeting” concept, a single-molecule NIR dye Ph790H could achieve tumor targeting, imaging, and therapy simultaneously. Hence, the present study suggests a simple but efficient strategy to design and synthesize the multifunctional phototheranostic agents for cancer-targeted diagnosis and therapy. At present, most of the in vivo studies are still dependent on small animals, and a certain gap exists in the actual realization of extensive clinical application. With the progress and development of photo-based diagnosis and treatment methods, the multi-mode combined treatment strategy can provide a new opportunity and challenge for the next generation of cancer therapy.

## Data Availability

Data are contained within the article.
